# RABV P protein activates the GSK3β-NF-κB signaling pathway to promote viral replication by interacting with the host protein Snapin

**DOI:** 10.1128/jvi.01006-26

**Published:** 2026-09-15

**Authors:** Yujie Bai, Yongsai Liu, Xiaoyu Wu, Hongli Jin, Zhiyuan Gong, Yumeng Song, Xingqi Liu, Zihang Gao, Zhenhong Guan, Pei Huang, Yuanyuan Li, Haili Zhang, Hualei Wang

**Affiliations:** 1 State Key Laboratory for Diagnosis and Treatment of Severe Zoonotic Infectious Diseases, Key Laboratory for Zoonosis Research of the Ministry of Education, Institute of Zoonosis, and College of Veterinary Medicine, Jilin University 12510, Changchun, China; University of Freiburg, Freiburg, Germany

**Keywords:** rabies virus, phosphoprotein, Snapin protein, GSK3β protein, NF-κB pathway

## Abstract

Rabies virus (RABV), a lethal neurotropic pathogen, relies on interaction with the host to sustain neuronal infection; however, how it balances replication with neuronal survival remains unclear. Here, we revealed Snapin, a neuronal trafficking regulator, to be a critical host factor co-opted by RABV to enhance neuroinvasion. We found that RABV infection upregulated Snapin expression in N2a cells, and Snapin could promote the replication of RABV CVS11 and SRV9 strains in a dose-dependent manner. Mechanistic studies demonstrated that RABV P protein interacts with the 37–78 aa domain of Snapin via its C-terminal domain (172–297 aa), recruits glycogen synthase kinase 3β (GSK3β) in a Snapin-dependent manner, and forms a tripartite complex. Upon complex formation, the phosphorylation level at the Tyr216 site of GSK3β is increased, leading to activation of the NF-κB pathway. This activation enhances viral replication by upregulating neuronal stability genes (*Septin2, B4gat1*, and *Disc1*) without inducing interferon responses. *In vivo* studies show that knockdown of Snapin in infected mice delays clinical symptom onset and improves host survival. This study uncovers a unique strategy wherein RABV exploits Snapin as a molecular scaffold linking P to GSK3β/NF-κB signaling, coupling viral replication to neuronal homeostasis. Our work identifies a host-dependent axis for therapeutic targeting in rabies and offers broader implications for understanding neurotropic pathogens utilizing analogous mechanisms.

**IMPORTANCE:**

Rabies virus (RABV) depends on host factors to maintain neuronal infection while avoiding cell death, but the mechanisms balancing viral replication and neuronal survival are unclear. This study identifies Snapin as a critical host factor commandeered by RABV to enhance neuroinvasion. Upon RABV infection, Snapin is upregulated and subsequently hijacked by the C-terminal domain of the RABV P protein. This interaction recruits GSK3β in a Snapin-dependent manner, forming a tripartite complex that increases phosphorylation of GSK3β at Tyr216 and activates the NF-κB pathway. Such activation upregulates neuronal stability genes (*Septin2*, *B4gat1*, and *Disc1*), thereby promoting viral replication without eliciting interferon responses. *In vivo* functional validation further confirms that Snapin deficiency attenuates RABV pathogenicity and prolongs host survival. Collectively, our study highlights a Snapin-dependent GSK3β/NF-κB axis as a potential therapeutic target for rabies treatment.

## INTRODUCTION

Rabies virus (RABV), a neurotropic virus belonging to the *Lyssavirus* genus of the *Rhabdoviridae* family, invariably causes fatal encephalitis, known as rabies, in both humans and warm-blooded animals. Given the absence of effective clinical treatments, the mortality rate of rabies reaches 100% once clinical symptoms manifest, posing a significant threat to public health and community safety. The neurotropism of RABV is characterized by its unique invasion strategy: following peripheral entry (e.g., via animal bites), the virus engages in retrograde axonal transport along motor neurons through dynein-mediated trafficking, thereby evading lysosomal degradation and ultimately reaching the central nervous system (CNS) within specialized transport vesicles (1). Healthy neurons have comprehensive cellular defense mechanisms that can initiate a resistance response to counter viral invasion, such as impeding viral replication via signaling pathways like the interferon pathway (2, 3). However, RABV has evolved diverse strategies to overcome these defenses through its structural proteins.

The RABV genome consists of a ~12-kb single-stranded negative-sense RNA encoding five structural proteins arranged 3′-N-P-M-G-L-5′. The nucleoprotein (N), phosphoprotein (P), and large polymerase (L) form a ribonucleoprotein (RNP) complex essential for transcription and replication. The trimeric glycoprotein (G), the sole viral surface protein, dictates neuropathogenicity by mediating receptor binding (e.g., neural cell adhesion molecule, NCAM) and tissue tropism (4–6). Notably, RABV G also functions as a critical switch that regulates neuronal survival versus apoptosis during early infection. Specifically, the PDZ-binding motif in its cytoplasmic tail dictates strain-specific outcomes: pathogenic strains express low levels of G to preserve neuronal integrity, thereby supporting sustained axonal transport and viral dissemination, whereas attenuated strains with altered G sequences induce robust neuronal apoptosis (3, 7). The matrix protein (M) orchestrates virion assembly and budding via RNP-host membrane interactions while concurrently suppressing the activation of the NLRP3 inflammasome and co-opting autophagosomes to enhance viral replication (8). The RABV P protein is a multifunctional protein, being not only indispensable for viral transcription and replication as a cofactor for the L protein, but also interacting with many cellular factors to help escape the host innate immune response. Additionally, the C-terminal domain of the P protein, particularly residues W265 and M287, is involved in the interaction with IFN-activated STAT1 (pSTAT1) and cytoplasmic retention of pSTAT1 to block IFN signaling (9).

Neurotropic viruses promote self-replication and transmission by regulating the structural integrity of host neurons at different stages of infection (10). Theiler’s murine encephalomyelitis virus (TMEV) exhibits enhanced pathogenicity when axonal degeneration is inhibited. Similarly, in the early stage of CVS11 infection in mice, there is minimal neuronal degeneration despite mild inflammation during the initial phases (11), suggesting that neurotropic viruses exploit neuronal stability for replication at the early stage of infection. Beyond the anti-apoptotic function of RABV M protein (12), accumulated evidence highlights a central regulatory role of the viral G protein in balancing neuronal viability (13, 14). However, the specific mechanisms by which RABV replicates within neurons and protects neuronal integrity in the early stages of infection remain to be elucidated.

Snapin, a SNARE-associated protein originally identified in neurons, localizes predominantly to synaptic vesicle membranes and modulates synaptic transmission through its interaction with the SNARE complex (15–17). Emerging evidence indicates that Snapin plays a pivotal role in maintaining neuronal homeostasis by orchestrating critical cellular processes, including late endosome-to-lysosome trafficking, lysosomal maturation, and autophagic flux (18–21). Beyond its physiological functions, accumulating studies highlight Snapin’s involvement in the replication cycles of diverse viruses through distinct molecular mechanisms (22–25). Snapin interacts with multiple human cytomegalovirus (HCMV)-encoded proteins (e.g., UL70, UL105, UL142, and UL130), modulating the intracellular localization of viral DNA replication enzymes and thereby affecting HCMV replication (26–28). In contrast, Snapin enhances HIV-1 infectivity by suppressing TLR8-mediated viral sensing in endosomal compartments, promoting immune evasion and interhost viral transmission (22). These findings collectively demonstrate that Snapin has a multifaceted role in viral pathogenesis, with mechanism-specific adaptations across different viral species. Given that RABV depends on axonal transport for CNS invasion, and our results found that RABV infection led to a significant upregulation of Snapin protein expression, we hypothesize that Snapin may serve as a critical host factor in RABV infection.

In this study, we found that the host protein Snapin positively regulates the replication of RABV. Further results revealed that the Snapin protein interacts with the RABV P protein, thereby mediating the interaction between the RABV P protein and the host factor GSK3β. This interaction activates the NF-κB signaling pathway by modulating the phosphorylation level of GSK3β at Tyr216, which subsequently promotes the expression of downstream neuron-related factors, thereby facilitating RABV replication. Taken together, these findings uncovered a cell-intrinsic function of Snapin for RABV infection and paved the way for developing rabies therapeutics.

## RESULTS

### Snapin facilitates RABV infection

**Fig 1:**
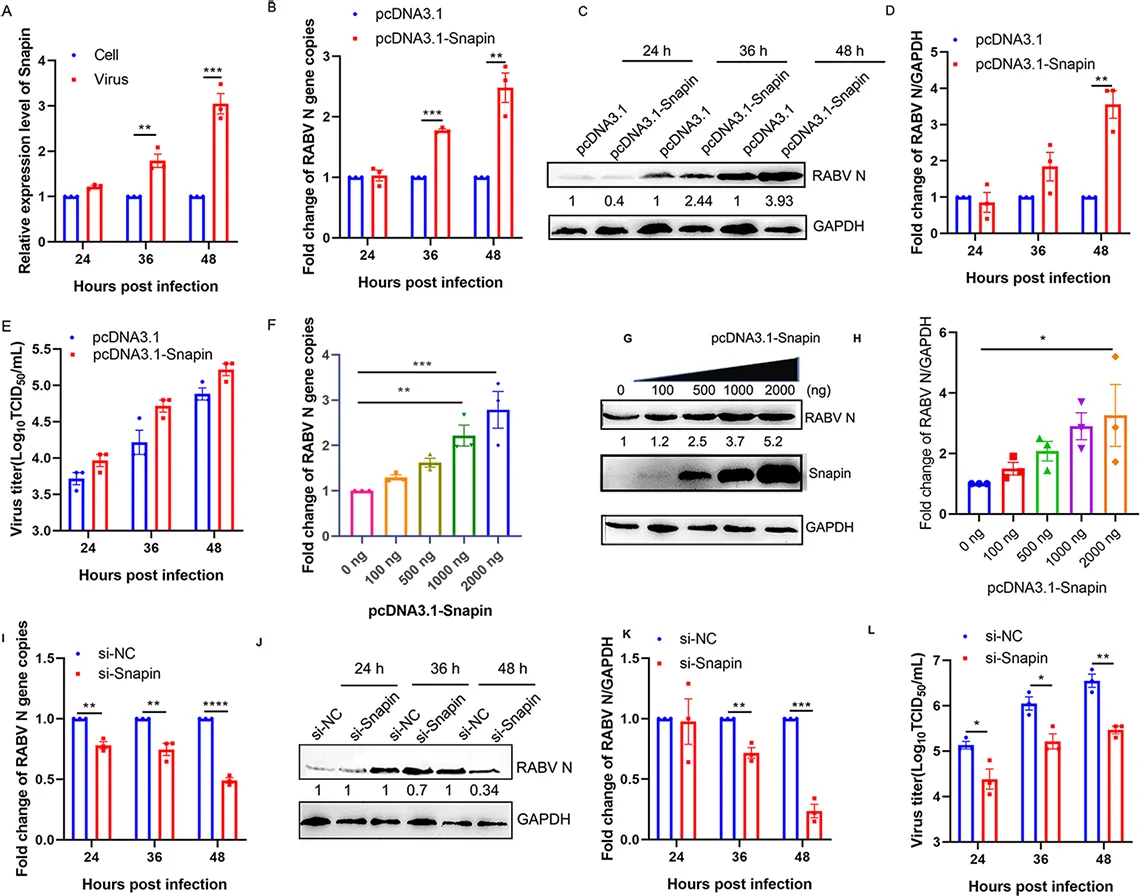
The host protein Snapin participates in RABV infection. (**A**) N2a cells were infected with RABV at MOI = 0.1 and collected at the indicated time points post-infection. The Snapin mRNA levels were determined using qPCR. (B–E) N2a cells were transfected with an empty vector or pcDNA3.1-Snapin for 24 h, followed by infection with RABV CVS11 (MOI = 0.1) for 24, 36, or 48 h. The levels of RABV *N* transcripts were measured using qPCR (**B**), and the RABV N protein expression levels were measured with western blot (WB) and analyzed with densitometric analysis using ImageJ, normalized to GAPDH (**C and D**). The virus titer was tested with the TCID_50_ assay (**E**). (F–H) N2a cells were transfected with either an empty vector or different doses of pcDNA3.1-Snapin for 24 h (the total transfection amount for each well was 2,000 ng. For the groups where Snapin did not reach 2,000 ng, an empty vector was used for supplementation), followed by infection with RABV (MOI = 0.1) for 36 h. The RABV *N* transcription levels were measured using qPCR (**F**), and the RABV N protein expression levels were measured with WB and analyzed with densitometric analysis using ImageJ, normalized to GAPDH (**G and H**). (**I**) N2a cells were transfected with si-NC or si-Snapin for 24 h and then infected with RABV (MOI = 0.1) for 24, 36, or 48 h. The levels of RABV *N* transcription were measured using qPCR. (**J and K**) The protein expression levels of RABV N were measured with WB (J) and analyzed with densitometric analysis using ImageJ, normalized to GAPDH (K). (**L**) The virus titers were determined using the TCID_50_ assay. All WB band grayscale analyses were performed using ImageJ software, normalized to GAPDH, and the experimental group was compared with its respective standardized control group. Data were expressed as means ± SEM of triplicate wells from one independent experiment. Statistical significance was determined using the Student’s *t*-test with a multiple comparison correction. *, *P* < 0.05, **, *P* < 0.01, ****, *P* < 0.0001.

To investigate the role of the host protein Snapin in RABV infection, we first examined its expression changes in mouse neuroblastoma Neuro-2a (N2a) cells following challenge with the RABV strain CVS11 at a multiplicity of infection (MOI) of 0.1. The quantitative real-time PCR (qPCR) results showed that RABV infection resulted in the upregulation of the Snapin mRNA expression levels at 36 and 48 h post-infection (hpi) (Fig. 1A). Next, the impact of the Snapin protein on the replication of RABV was evaluated in N2a cells. The results showed that the overexpression of Snapin significantly increased the viral mRNA expression levels (Fig. 1B), levels of the viral protein (Fig. 1C and D), as well as the viral titers (Fig. 1E) of RABV CVS11 at 36 and 48 hpi, and this enhancing effect was dose-dependent (Fig. 1F through H). To identify whether knockdown of Snapin had the opposite effect on RABV infection, small interfering RNAs (siRNAs) specifically targeting the *Snapin* gene were designed, and the knockdown efficiency was confirmed using qPCR (Fig. S1A). As shown in Fig. 1I through L, compared to the control group, the viral mRNA levels (Fig. 1I), the protein expression levels (Fig. 1J and K), and the viral titers (Fig. 1L) of RABV CVS11 were markedly inhibited at 36 and 48 hpi in the Snapin-knockdown group. Subsequently, to investigate whether the effect of Snapin on RABV was virus strain-specific, we tested the impact of Snapin on the RABV vaccine strain SRV9. The results demonstrated that the overexpression of Snapin significantly increased the viral mRNA and the viral protein expression levels of RABV SRV9 at 36 hpi, while the opposite trend was observed after the knockdown of Snapin (Fig. S1B and C). These results indicated that the host protein Snapin plays an important role in RABV replication.

### The host protein Snapin interacts with the RABV P protein

**Fig 2:**
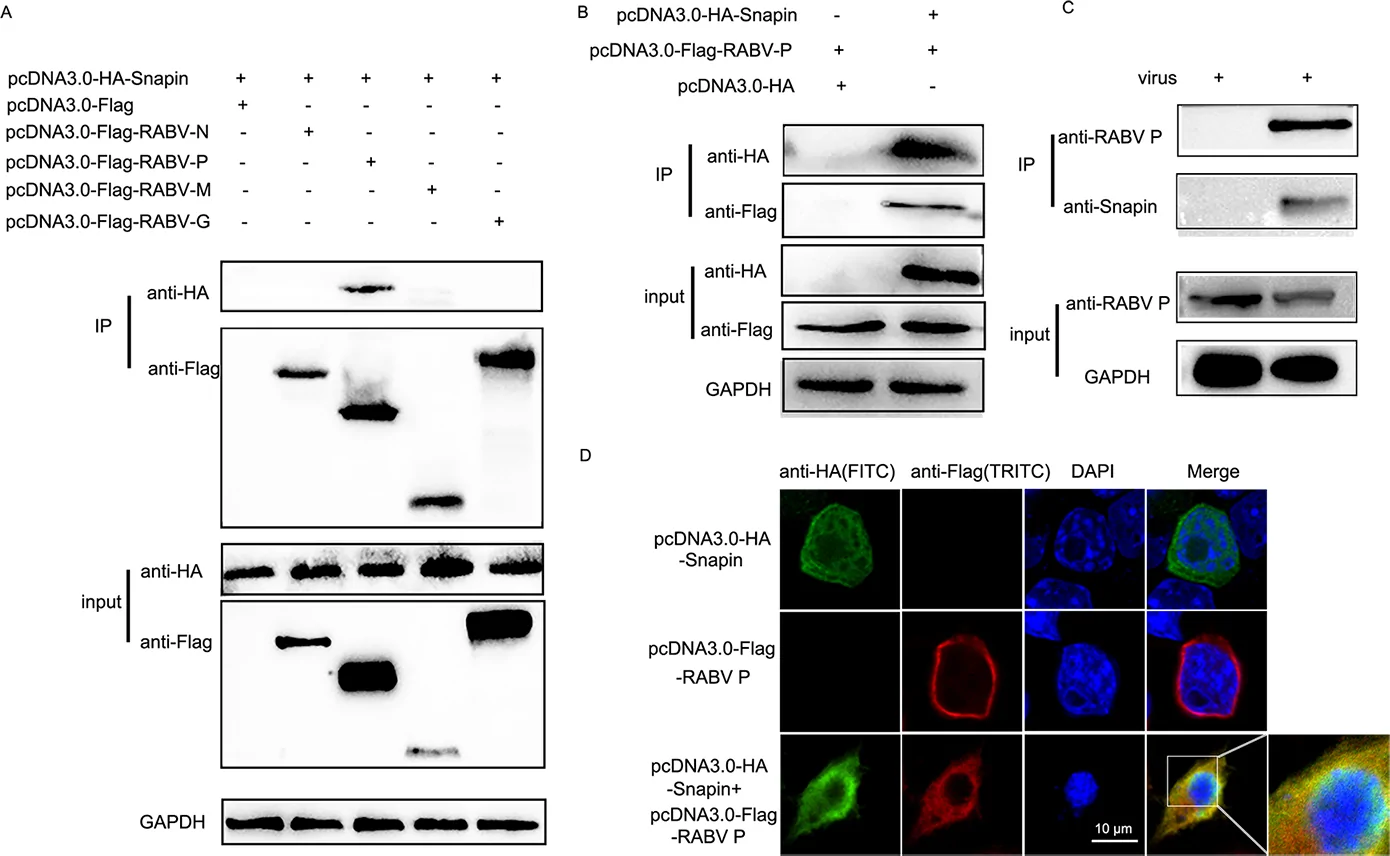
The host protein Snapin interacts with RABV P protein. (**A**) Lysates of HEK293T cells transfected with the indicated plasmids (HA-tagged Snapin and Flag-tagged RABV structural protein) were subjected to anti-Flag immunoprecipitation and analyzed using WB. (**B**) Lysates of HEK293T cells transfected with the indicated plasmids (HA-tagged Snapin or HA vector and Flag-tagged RABV P) were subjected to anti-HA immunoprecipitation and analyzed using WB. (**C**) Lysates of N2a cells infected with the RABV CVS11 strain were immunoprecipitated with anti-Snapin or control IgG, followed by WB analysis. (**D**) HEK293T cells were co-transfected with HA-tagged Snapin or HA vector and Flag-tagged RABV P. After 36 h, the cells were immunostained with anti-Flag antibody (red) and anti-HA antibody (green). Scale bar: 10 μm.

To elucidate the mechanism by which Snapin affects RABV replication, we employed a co-immunoprecipitation (Co-IP) assay to determine whether Snapin interacts with the RABV structural proteins N, P, M, or G. The HA-tagged Snapin and Flag-tagged RABV structural proteins were co-transfected into HEK293T cells, and the cell lysates were analyzed using Co-IP assays with anti-Flag beads. As shown in Fig. 2A, only RABV P protein interacted with Snapin. The reverse Co-IP assays were performed using anti-HA beads, and the results showed that Snapin could interact with RABV P (Fig. 2B). Under viral infection, the Co-IP results revealed that the endogenous Snapin protein could interact with the P protein of RABV (Fig. 2C). Moreover, confocal microscopy suggested that Snapin co-localized with RABV P in the cytoplasm (Fig. 2D). We also verified the interaction between the host protein Snapin and the P proteins of different virus strains (CVS11, SRV9, and DRV) through Co-IP experiments. The results showed that Snapin interacts with the P proteins of all three tested virus strains (Fig. S2).

**Fig 3:**
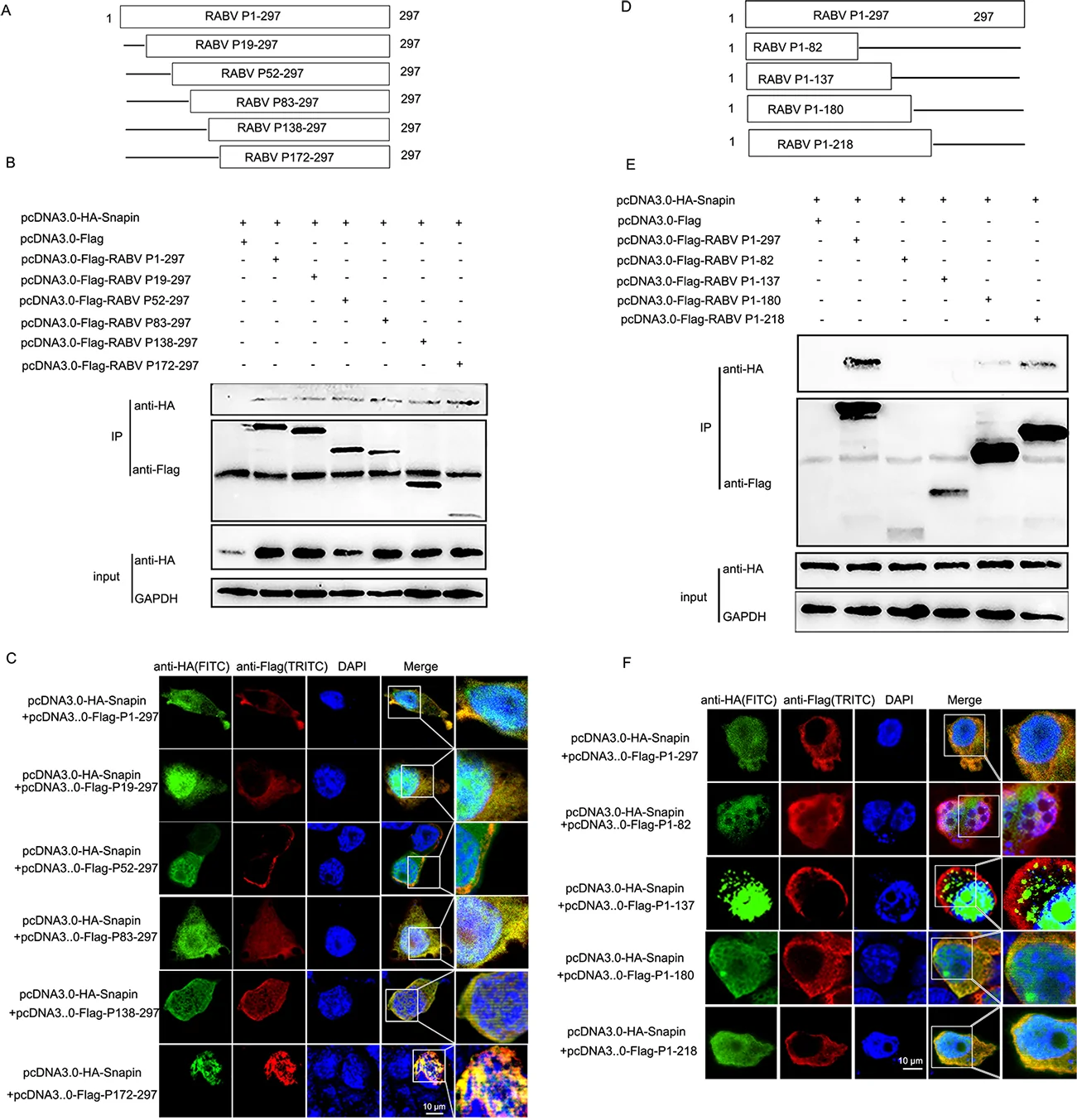
The host protein Snapin interacts with the C-terminal domain of RABV P protein. (**A**) Full-length P protein and N-terminal truncated P protein are shown in the scheme. (**B**) HEK293T cells were transiently transfected with the full-length or truncated versions of RABV P, followed by IP and WB analyses to determine the domains crucial for the Snapin-RABV P interaction. (**C**) HEK293T cells were co-transfected with HA-tagged Snapin and truncated RABV P expression vectors for 36 h, fixed, and immunostained with mouse anti-Flag antibodies (red) and anti-HA antibodies (green), and then visualized using confocal microscopy. DAPI (blue) was used to stain nuclear DNA. (**D**) Full-length P protein and C-terminal truncated P protein are shown in the scheme. (**E**) HEK293T cells were transiently transfected with the full-length or truncated versions of RABV P, followed by IP and WB analyses to determine the domains crucial for the Snapin-RABV P interaction. (**F**) HEK293T cells were co-transfected with HA-tagged Snapin and truncated RABV P expression vectors for 36 h, fixed, and immunostained with mouse anti-Flag antibodies (red) and anti-HA antibodies (green), and then visualized using confocal microscopy. DAPI (blue) was used to stain nuclear DNA. Scale bar: 10 μm.

To explore the molecular details of the P-Snapin interaction, five N-terminal truncated P mutants were constructed according to the naturally existing P variants and their function domains (29, 30): RABV P19-297 (P2), RABV P52-297 (P3), RABV P83-297 (P5), RABV P138-297, and RABV P172-297 (Fig. 3A). Subsequently, the P N-terminal mutants were co-transfected with HA-tagged Snapin in HEK293T cells and analyzed using Co-IP and confocal assays. The results showed that all these P N-terminal mutants were able to interact with Snapin (Fig. 3B) and that they were also able to co-localize with Snapin in the cytoplasm (Fig. 3C). Then, the C-terminal truncated P mutants were constructed to further determine the specific region of the RABV P protein responsible for the interaction with Snapin (Fig. 3D). Subsequently, the P C-terminal mutants were co-transfected with HA-tagged Snapin in HEK293T cells and analyzed using Co-IP and confocal assays. The Co-IP results indicated that P1–218 and P1–180 were able to interact with Snapin, while P1–82 and P1–137 were not. Moreover, P truncates with longer C-terminal sequences were more capable of interacting with Snapin protein (Fig. 3E). The confocal microscopy results also indicated that P1–218 and P1–180 could co-localize with Snapin protein in the cytoplasm (Fig. 3F). All these results suggested that the C terminal of the RABV P protein, especially the 172–218 aa region, was critical for its interaction with Snapin.

### The 37–78 aa of Snapin is crucial for the interaction with the RABV P protein and promotion of viral replication

**Fig 4:**
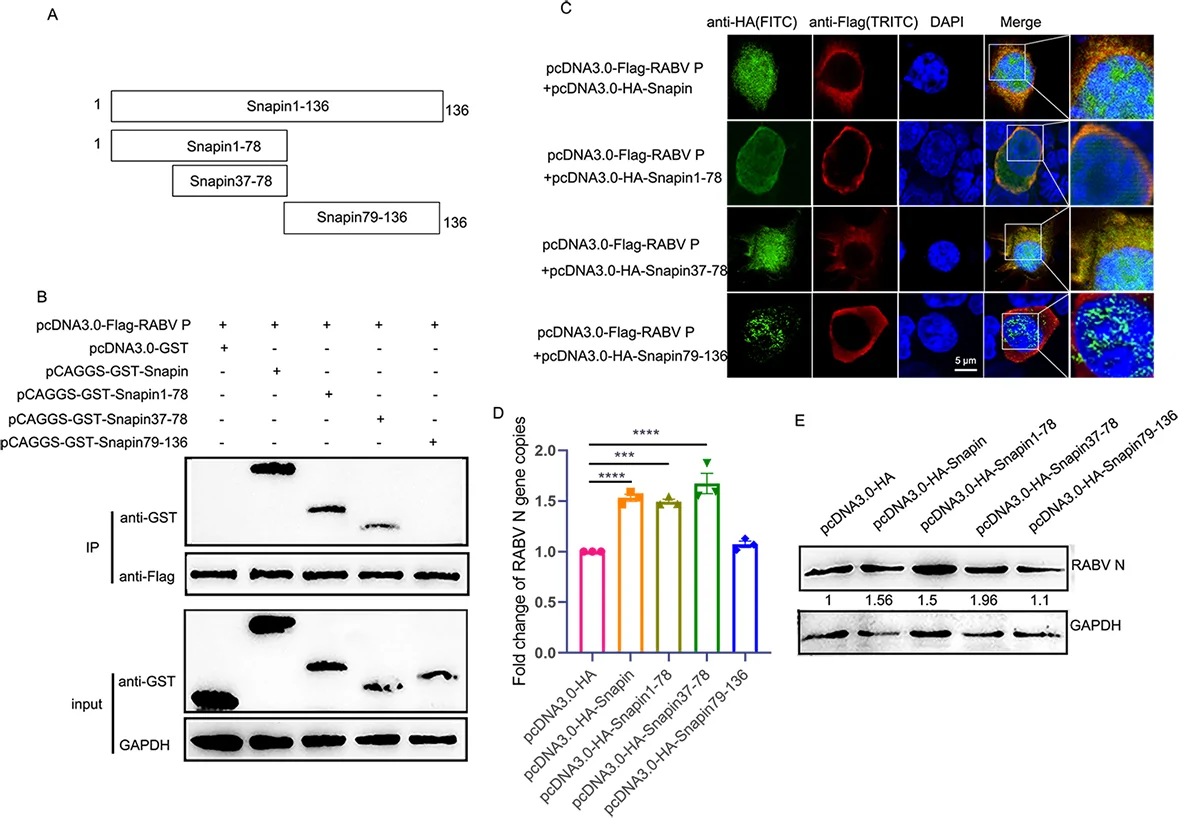
The 37–78 aa of Snapin is crucial for the interaction with RABV P protein and for promoting viral replication. (**A**) Schematic diagram of the truncated mutants of the Snapin protein, constructed according to the known domains. (**B**) HEK293T cells were co-transfected with RABV P and the full-length or truncated mutants of Snapin. After 36 h, the cells were lysed and analyzed using Co-IP with anti-Flag nanomagnetic beads. (**C**) HEK293T cells were co-transfected with RABV P and the full-length or truncated mutants of Snapin. After 36 h, the cells were immunostained with mouse anti-Flag antibodies (red) and anti-HA antibodies (green), and then observed by confocal microscopy. DAPI (blue) was used to stain nuclear DNA. Scale bar: 5 μm. (**D and E**) N2a cells were transfected with the expression plasmids of full-length or truncated mutants of Snapin for 24 h and were then infected with RABV (MOI = 0.1) for 36 h. The transcription levels of RABV *N* were measured using qPCR (D). The RABV N protein expression levels were measured using WB (E). All WB band grayscale analyses were performed using ImageJ software, normalized to GAPDH, and the experimental group was compared with its respective standardized control group. Data were expressed as means ± SEM of triplicate wells from one independent experiment. Statistical significance was determined by the Student’s *t*-test with a multiple comparison correction. ***, *P* < 0.001.

Structurally, Snapin mainly consists of a transmembrane hydrophobic region (1–20 aa) in the N-terminus and a coiled-coil formed by two spiral regions, H1 and H2, in the C-terminus. This coiled-coil domain is highly conserved and is functionally associated with vesicle fusion proteins, as previously reported. To investigate the domain of the Snapin protein key for the interaction with RABV P, three Snapin truncated mutants were constructed according to the known structure: Snapin1–78, Snapin37–78, and Snapin79–136 (Fig. 4A). Subsequently, HEK293T cells were co-transfected with Flag-tagged RABV P and GST-tagged wild-type (WT) or truncated Snapin expression plasmids. The Co-IP and confocal results showed that both the Snapin1–78 and Snapin37–78 mutants interacted with the RABV P protein (Fig. 4B and C).

To further elucidate the key functional domains of the Snapin protein necessary for the support of RABV replication, the full-length Snapin and its truncated mutants were transfected into N2a cells, followed by infection with RABV CVS11 (MOI = 0.1) at 24 h post-transfection. The qPCR and WB results showed that truncated mutants Snapin1–78 and Snapin37–78 showed the same level of support for RABV replication as did the full-length Snapin protein (Fig. 4D and E). All these results indicated that the 37–78 aa of Snapin was crucial for its function in interacting with RABV P protein and promoting viral replication.

### Snapin promotes RABV replication by interacting with the P protein to activate the NF-κB pathway and downstream gene expression

**Fig 5:**
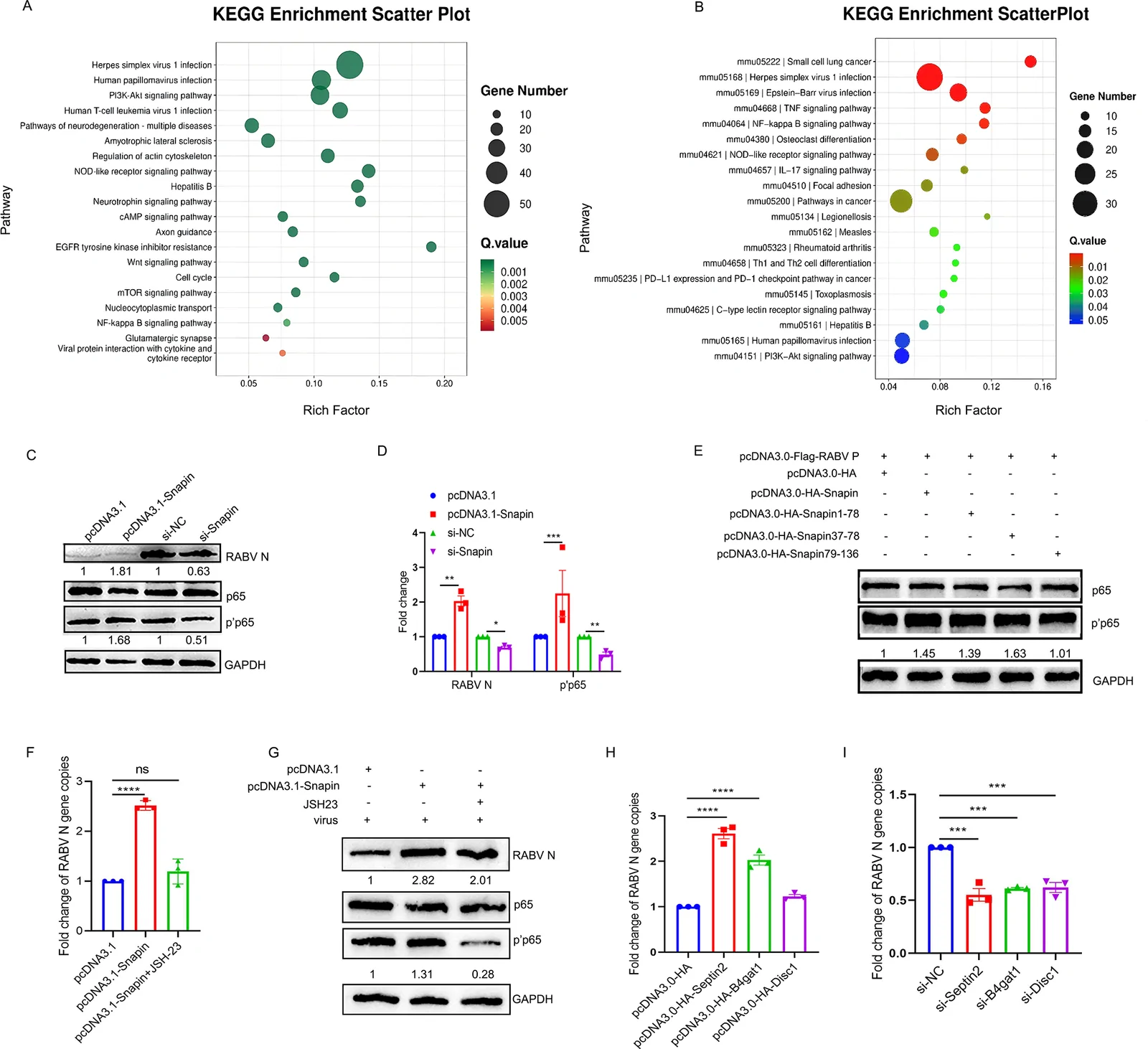
The host factor Snapin regulates RABV replication through the NF-κB signaling pathway. (**A and B**) N2a cells were transfected with an empty vector, pcDNA3.1-Snapin, si-NC, or si-Snapin for 24 h, then infected with RABV CVS11 (MOI = 0.1). Cells were collected after 36 h, and RNA was extracted using the TRIzol method for KEGG analysis. (**C and D**) N2a cells were transfected with an empty vector or pcDNA3.1-Snapin for 24 h, followed by infection with RABV CVS11 (MOI = 0.1) for 24, 36, or 48 h. The levels of RABV N protein and phospho-NF-κB-p65 protein were measured using WB. (**E**) N2a cells were co-transfected with full-length Snapin, its truncated mutants, and the RABV P protein, followed by western blot analysis to determine the phosphorylation level of NF-κB-p65. (**F and G**) N2a cells were transfected with an empty vector or pcDNA3.1-Snapin for 24 h, followed by infection with RABV CVS11 (MOI = 0.1) for 18 h and then treated with JSH-23 for 6 h. The levels of RABV *N* transcription were measured using qPCR (F). The levels of RABV N protein and the phospho-NF-κB-p65 protein were measured using WB (G). (**H and I**) N2a cells were transfected with an empty vector, pcDNA3.0-HA-Septin2, pcDNA3.0-HA-B4gat1, pcDNA3.0-HA-Disc1, si-NC, si-Septin2, si-B4gat1, or si-Disc1 for 24 h, followed by infection with RABV CVS11 (MOI = 0.1) for 36 h. The levels of RABV *N* transcription were measured using qPCR (H). The levels of RABV N protein and the phospho-NF-κB-p65 protein were measured using WB (I). All WB band grayscale analyses were performed using ImageJ software and normalized to GAPDH, and the experimental group was compared with its respective standardized control group. Data were expressed as means ± SEM of triplicate wells from one independent experiment. Statistical significance was determined using Student’s *t*-tests with a multiple comparison correction. *, *P* < 0.05, **, *P* < 0.01, ***, *P* < 0.001.

Previous research has demonstrated that the C-terminal domain (CTD) of the P protein plays a crucial role in inhibiting the interferon (IFN) response by interacting with key host proteins involved in the IFN pathway, including STAT1, 2, and 3, as well as the PML protein. Given that the C-terminal region of the P protein mediates its interaction with Snapin, it is hypothesized that Snapin is implicated in regulating the host IFN response during RABV infection. We detected the expression levels of IFN downstream factors during RABV infection with or without the overexpression of Snapin. The qPCR results showed that during RABV infection, the overexpression of Snapin did promote RABV replication but did not cause any changes in the expression of *IFN-β*, *STAT1*, *ISG15*, or *IRF3*, suggesting that the IFN response was not the main pathway for Snapin-mediated promotion of RABV replication (Fig. S3A). To further explore the mechanism by which Snapin regulates RABV replication, N2a cells were transfected with pcDNA3.1, pcDNA3.1-Snapin, siNC, or siSnapin for 24 h. The cells were then infected with RABV at an MOI of 0.1 for 36 h. The above samples were collected and sent for RNA-sequencing (RNA-seq) analysis by the Lianchuan Company, and the differentially expressed genes (DEGs) were mapped to typical signaling pathways in KEGG. The results showed that in both the Snapin overexpression group and the knockdown group, the DEGs were both enriched in the NF-κB pathway and PI3K-Akt pathway. The DEGs common to both the overexpression and knockdown groups were further subjected to Gene Ontology (GO) enrichment analysis, and most DEGs were found to be involved in the NF-κB signaling pathway (Fig. 5A and B).

To investigate whether Snapin promotes RABV replication by regulating the NF-κB pathway, we first examined the effect of Snapin on the NF-κB pathway. Our results showed that neither overexpression nor knockdown of Snapin caused significant changes in the NF-κB pathway (Fig. S3B). Interestingly, during RABV infection, the overexpression of Snapin promoted viral replication and activated the NF-κB signaling pathway, while downregulation of Snapin resulted in the opposite trend (Fig. 5C and D). These results suggested that the activation of the NF-κB signaling pathway was the result of the co-action of the host protein Snapin together with RABV infection. We next verified whether the regulation of the NF-κB pathway by Snapin during RABV infection was related to the viral P protein. The pcDNA3.0-Flag-RABV P plasmid was co-transfected into N2a cells together with full-length and truncated forms of Snapin. Western blot analysis revealed that the minimal interaction region of Snapin with the P protein, like the full-length form, significantly enhanced NF-κB pathway activation (Fig. 5E). These results indicate that Snapin activates the NF-κB signaling pathway in an interaction-dependent manner with the RABV P protein. Then, we introduced an NF-κB pathway inhibitor (JSH-23) to further confirm the crucial role of the Snapin-regulated NF-κB signaling pathway during RABV infection. The N2a cells were transfected with either pcDNA3.1-Snapin or an empty vector and then infected with RABV CVS11 (MOI = 0.1) at 24 h post-transfection. At 18 h post-infection, the cells were incubated with 50 μM JSH-23 for 6 h (31). The qPCR and WB results showed that the overexpression of Snapin promoted RABV replication at both the mRNA (Fig. 5F) and protein levels (Fig. 5G) and activated the NF-κB signaling pathway, while the treatment with JSH-23 simultaneously inhibited viral replication and the Snapin-induced NF-κB pathway. Together, these data suggest that the Snapin protein activates the NF-κB signaling pathway by interacting with the RABV P protein, which, in turn, promotes RABV replication.

Given that the NF-κB signaling pathway has been recognized as a crucial pathway in modulating inflammatory responses, we next examined the changes in inflammatory response-related factors following RABV infection in cells in which Snapin was either overexpressed or knocked down. The qPCR results showed that the *Snapin* expression levels were positively associated with the extent of viral replication, but not with the expression of downstream inflammatory factors (Fig. S4A), indicating that the Snapin-mediated NF-κB signaling pathway did not exert its impact on RABV replication through the modulation of downstream inflammatory factors and chemokines. Then, we compared up- and down-regulated DEGs from RNA-seq in the Snapin-overexpression and Snapin-knockdown groups and identified 268 candidate genes (Fig. S4B). We further analyzed selected high-confidence genes, as shown in Fig. S4C, and found that the candidate genes, including *Disc1*, *B4gat1*, and *Septin2*, were mainly related to neuronal development and stability (32–37). Subsequently, we confirmed whether these genes were regulated by the Snapin-mediated NF-κB pathway during RABV infection. The qPCR results demonstrated that overexpression of *Snapin* enhanced the expression of these genes, while JSH-23 treatment inhibited Snapin-induced expression of these genes (Fig. S4D). We further investigated the impact of Disc1, B4gat1, and Septin2 on RABV replication through loss- and gain-of-function experiments and qPCR. The results demonstrated that the overexpression of these host proteins significantly enhanced viral replication (Fig. 5H), whereas knockdown of these host proteins markedly suppressed viral replication (Fig. 5I). All these data suggested that Snapin interacts with RABV P protein to activate the NF-κB pathway and induce the expression of downstream host proteins associated with neuronal development and stability, thereby facilitating RABV replication.

### The Snapin/P complex regulates the NF-κB pathway by interacting with GSK3β and regulating its phosphorylation state

**Fig 6:**
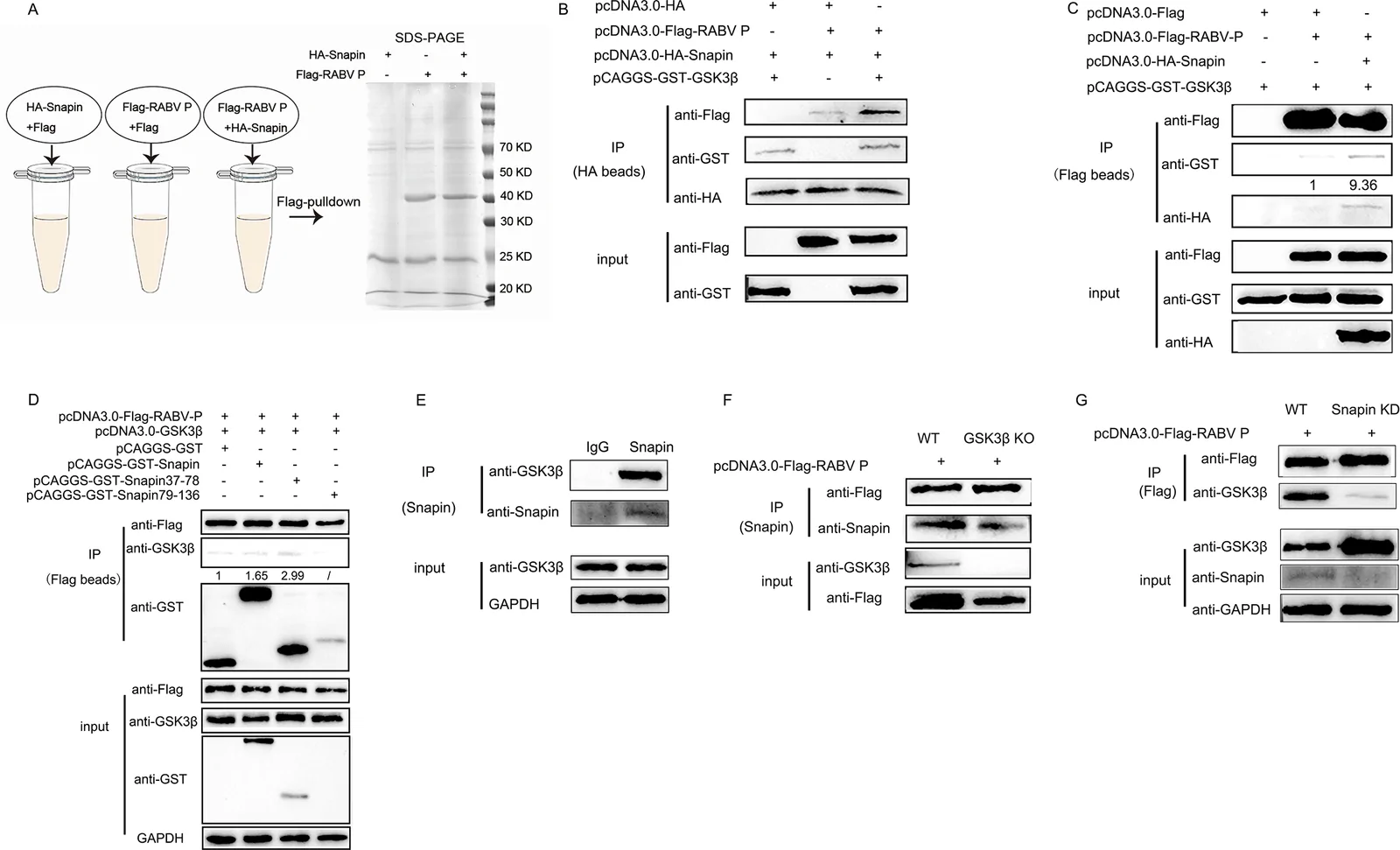
GSK3β interacts with the host protein Snapin and RABV P protein. (**A**) N2a cells were transfected with Snapin alone or together with RABV P protein, and cell samples were collected after 36 h. After lysis, the samples were enriched and eluted using Flag magnetic beads, followed by separation using SDS-PAGE and subsequent mass spectrometry analysis. (**B**) Lysates of N2a cells transfected with the indicated plasmids (HA-tagged Snapin, Flag-tagged RABV P protein, and GST-tagged GSK3β) were subjected to anti-HA immunoprecipitation and analyzed with WB. (**C**) Lysates of N2a cells transfected with the indicated plasmids (HA-tagged Snapin, Flag-tagged RABV P protein, and GST-tagged GSK3β) were subjected to anti-Flag immunoprecipitation and analyzed with WB. (**D**) Lysates of N2a cells transfected with designated plasmids (GST-tagged Snapin and its truncation mutants, Flag-tagged RABV P protein, and GSK3β) were subjected to anti-Flag immunoprecipitation, followed by WB analysis. (**E**) N2a cells were lysed and enriched with murine IgG or Snapin antibodies, followed by treatment with GSK3β antibodies. (**F**) The WT cells and GSK3β KO cells were transfected with pcDNA3.0-Flag-RABV P. The cell samples were lysed and enriched with Snapin antibodies, followed by treatment with Flag antibodies. (**G**) The WT cells and Snapin KD cells were transfected with pcDNA3.0-Flag-RABV P. The cell samples were lysed and enriched with Flag antibodies, followed by treatment with GSK3β antibodies. All WB band grayscale analyses were performed using ImageJ software and normalized to GAPDH, and the experimental group was compared with its respective standardized control group.

We have demonstrated that Snapin activates the NF-κB pathway through its interaction with P protein, thereby facilitating RABV replication. However, the mechanism by which the Snapin/P complex regulates the NF-κB pathway remains unknown. To address this question, the N2a cells were transfected with either pcDNA3.0-HA-Snapin or pcDNA3.0-Flag-RABV P or co-transfected with both plasmids (Fig. 6A). After 48 h, the cell lysates were collected, and immunoprecipitation mass spectrometry (IP-MS) was conducted to detect any potential host proteins interacting with the Snapin/P complex. A total of 236 potential host proteins were identified. Of these, two host proteins, glycogen synthase kinase 3-β (GSK3β) and S100B, were related to the NF-κB signaling pathway (Fig. S5A). Co-IP assays confirmed that GSK3β had strong interactions with both the Snapin and RABV P, while S100B did not interact with either Snapin or RABV P (Fig. S5B). Moreover, GSK3β enhanced the interaction between the Snapin and RABV P protein (Fig. 6B), and the minimal interaction region of Snapin that interacts with the RABV P protein also enhances the interaction between GSK3β and RABV P (Fig. 6C and D). An endogenous Co-IP assay was then performed, and the results showed that there was an endogenous interaction between the two host proteins, GSK3β and Snapin (Fig. 6E). To further confirm the interaction details of this heterotrimer, we used a CRISPR-Cas9 system to separately construct *Snapin* knockout and *GSK3β* knockout cell lines. WB analysis demonstrated that the GSK3β knockout (GSK3β KO) cell line was constructed successfully, while Snapin was only partially knocked down (Fig. S5C and D). Next, the recombinant plasmid pcDNA3.0-Flag-RABV P was transfected into both wild-type (WT) and GSK3β KO cells. A Co-IP assay was performed using specific antibodies against Snapin combined with Protein A/G. The results showed that the endogenous Snapin was able to interact with RABV P protein even in GSK3β knockout cells, indicating that the interaction between Snapin and RABV P was not dependent on GSK3β protein (Fig. 6F). Then, to confirm the role of Snapin in this heterotrimer, the recombinant plasmids pcDNA3.0-Flag-RABV P and pCAGGS-GST-GSK3β were co-transfected into WT or Snapin knockdown (Snapin-KD) cells. Co-IP analysis demonstrated that the interaction between GSK3β and RABV P protein was significantly weakened in Snapin-KD cells (Fig. 6G). We obtained similar results regarding the interaction between endogenous GSK3β and RABV P protein in siSnapin cells (Fig. S5E). These findings indicate that these three proteins are able to form heterotrimers, that the Snapin protein mediates the interaction between GSK3β and the P protein, but that the interaction between the Snapin and RABV P protein does not depend on GSK3β.

**Fig 7:**
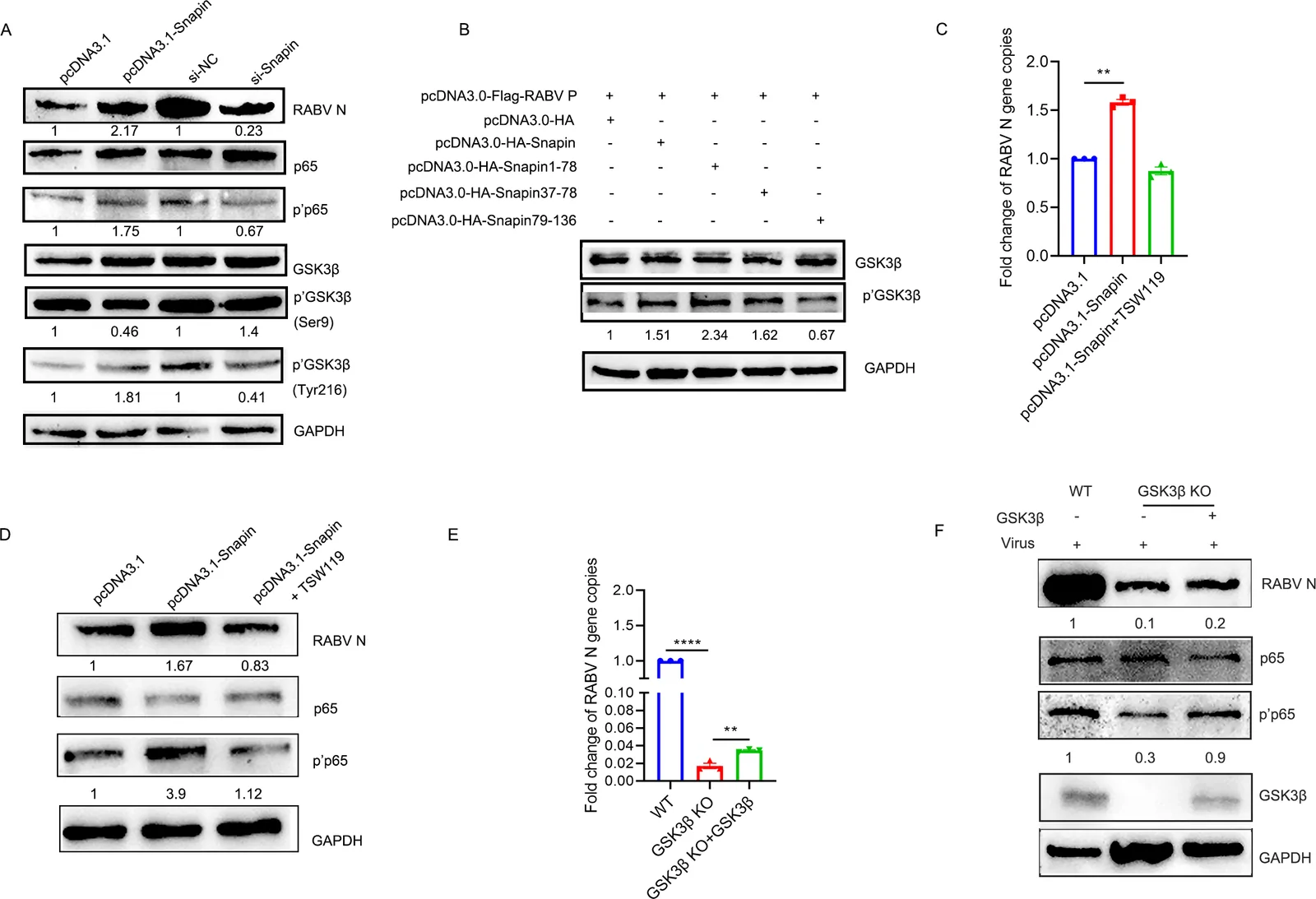
The host protein GSK3β plays an important role in RABV infection. (**A**) Effects of Snapin overexpression or knockdown on the NF-κB signaling pathway and GSK3β after RABV infection. N2a cells were transfected with an empty vector, pcDNA3.1-Snapin, si-NC, or si-Snapin for 24 h and then infected with RABV CVS11 (MOI = 0.1). The expression levels of RABV N protein, phosphorylation of P65, and phosphorylation of GSK3β were determined by WB. (**B**) N2a cells were co-transfected with full-length Snapin, its truncated mutants, and the RABV P protein, followed by western blot analysis to determine the phosphorylation level of GSK3β. (**C and D**) N2a cells were transfected with an empty vector or increasing amounts of Snapin-expressing plasmids for 24 h, followed by infection with RABV (MOI = 0.1) for an additional 30 h, and were then treated with TSW119, a GSK3β inhibitor, at a concentration of 100 µg/µL for 6 h. The levels of RABV N transcripts were measured using qPCR in collected N2a cells (C), while the RABV N protein and phospho-NF-κB-p65 protein levels were analyzed with WB (D). (**E and F**) The GSK3β knockout cells were transfected with a plasmid to rescue GSK3β expression for 24 h, followed by infection with RABV (MOI = 0.1) for an additional 36 h. The transcription levels of RABV N were measured using qPCR in collected N2a cells (E), while the protein levels of RABV N were analyzed through WB (F). All WB band grayscale analyses were performed using ImageJ software and normalized to GAPDH, and the experimental group was compared with its respective standardized control group. Data were expressed as means ± SEM of triplicate wells from one independent experiment. Statistical significance was determined using Student’s *t*-tests with a multiple comparison correction. **, *P* < 0.01, ***, *P* < 0.001.

It has been reported that GSK3β, one of the GSK3 isoforms, regulates the NF-κB signaling pathway through phosphorylation of its own tyrosine and serine/threonine. The phosphorylation at Ser9 is associated with the inhibition of both GSK3β kinase activity and the NF-κB signaling pathway, while phosphorylation of tyrosine at position 216 correlates with enhanced kinase activity and the activation of the NF-κB signaling pathway (38, 39). To investigate whether Snapin/P regulates the NF-κB signaling pathway by affecting the phosphorylation modification sites of GSK3β during RABV infection, N2a cells were transfected with the recombinant plasmid pcDNA3.1-Snapin or si-Snapin and then infected with the RABV CVS11 strain. The WB results showed that during RABV infection, the overexpression of Snapin enhanced virus replication and increased the phosphorylation levels of NF-κB p65 and GSK3β at Tyr216, while decreasing the phosphorylation levels of GSK3β at Ser9. Knockdown of Snapin during RABV infection showed the reverse effect, reducing virus replication, decreasing phosphorylation levels of NF-κB p65 and GSK3β at Tyr216, and increasing phosphorylation of GSK3β at Ser9 (Fig. 7A). We further demonstrate that this minimal P-interacting fragment also effectively promotes the phosphorylation levels of both GSK3β at Tyr216, comparable to the full-length Snapin (Fig. 7B). These results indicate that the Snapin37-78 fragment is not only sufficient for P-protein binding and viral replication enhancement, but also fully competent in activating the GSK3β/NF-κB signaling axis, thereby supporting the operative nature of the tripartite complex mechanism.

Then, we confirmed whether the function of Snapin in regulating the GSK3β/NF-κB pathway is associated with the RABV P protein. As shown in Fig. S6, RABV P protein could significantly activate the phosphorylation levels of GSK3β at Tyr216 and NF-κB p65 when Snapin was overexpressed. Conversely, RABV P protein was able to significantly inhibit the phosphorylation levels of GSK3β at Tyr216 and NF-κB p65 when Snapin expression was knocked down. These results indicate that Snapin and P protein co-regulate the downstream NF-κB signaling pathway by regulating the phosphorylation state of GSK3β.

To determine the role of GSK3β in Snapin-mediated promotion of RABV replication, a specific inhibitor of GSK3β, TWS119, was employed. N2a cells were transfected with pcDNA3.1-Snapin and infected with RABV CVS11 (MOI = 0.1). At 18 h following initial infection, 100 μM TWS119, which showed no cytotoxicity toward N2a cells, was added, and the infected cells were incubated for 6 h (Fig. S7). Samples were then collected and analyzed using qPCR and WB. The results showed that TWS119 treatment was able to suppress the Snapin-mediated promotion of viral replication effectively (Fig. 7C and D). We further validated the role of GSK3β in RABV replication through loss- and gain-of-function experiments. The qPCR and WB results demonstrated that knockout of GSK3β suppressed viral replication and the phosphorylation of NF-κB p65 significantly, while the reconstitution of GSK3β partially restored viral replication and phosphorylation levels of NF-κB p65 (Fig. 7E and F). These results indicate that GSK3β is indispensable to Snapin in the mediation of RABV replication through the NF-κB pathway.

### The host protein Snapin significantly enhances RABV replication *in vivo*

**Fig 8:**
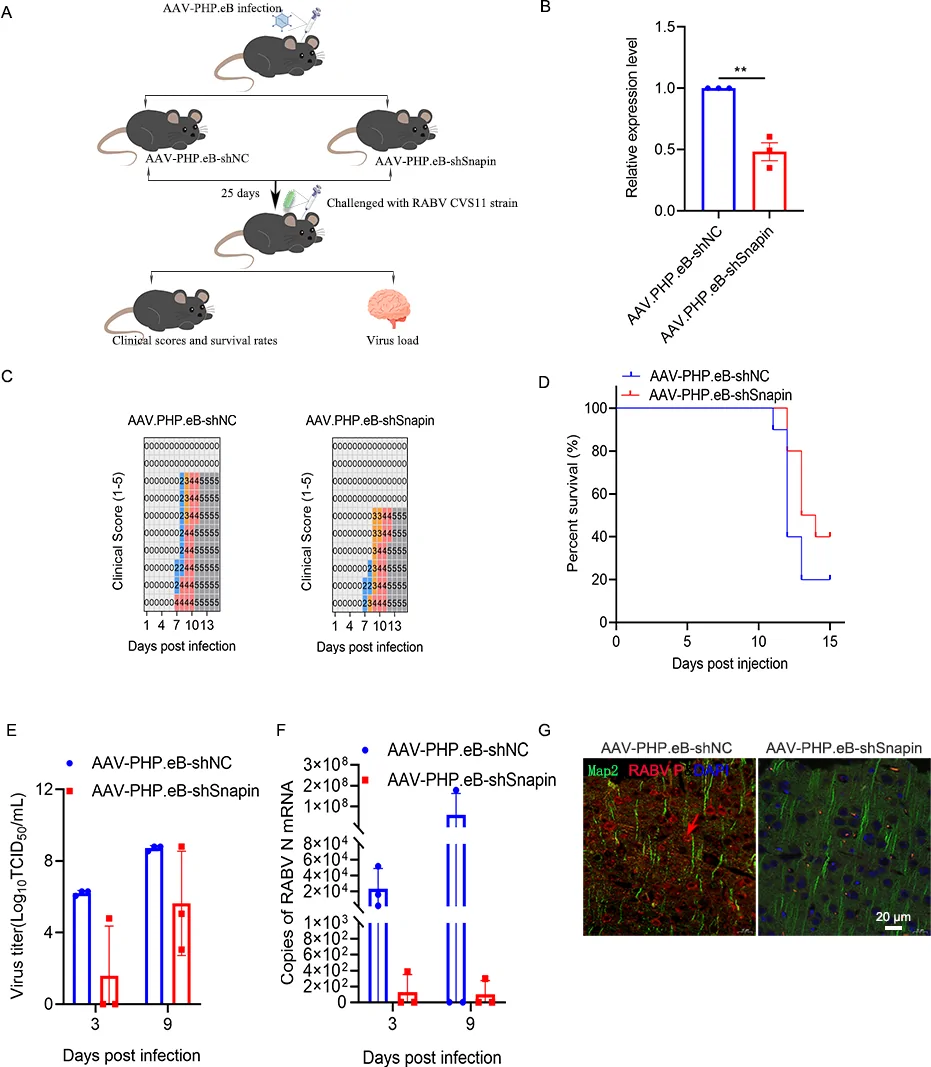
Effect of host protein Snapin knockdown on RABV replication *in vivo*. (**A**) Schematic representation of the construction of *Snapin* knockdown mice. (**B**) After 25 days of cerebral infection with 2 × 10^10^ vg/25 μL AAV-PHP.eB-shSnapin, qPCR was performed to assess Snapin mRNA expression levels in mouse brains. (**C and D**) The Snapin knockdown mice and control mice were intracranially infected with 10^1^ FFU of CVS11. The clinical symptoms (C) and survival rates (D) were monitored over a 15-day period. (**E and F**) Following intracranial infection with 10^1^ FFU of CVS11, brain tissues were collected from Snapin knockdown and control mice at the indicated time points. Viral titers were determined using the TCID_50_ assay, and (E) the transcription levels of the RABV *N* gene were quantified by qPCR (F). (**G**) IF analysis was conducted on brain tissues collected at 9 days post-infection. Brain sections were immunostained with mouse anti-RABV P antibodies (red), MAP2 (green), and DAPI (blue) for nuclear staining. Data were expressed as means ± SEM of triplicate wells from one independent experiment. Statistical significance was assessed using Student’s *t*-tests with a multiple comparison correction. **, *P* < 0.01.

To investigate the role of Snapin in RABV infection *in vivo*, we constructed a Snapin-knockdown mouse model by site-directed injection of 2 × 10^10^ vg/25 μL of AAV-PHP.eB-shSnapin into the mouse brain (Fig. 8A). The knockdown efficiency was verified by qPCR 25 days post-injection (Fig. 8B). The control mice were injected with AAV-PHP.eB-shNC. Subsequently, both control mice and Snapin-knockdown mice were intracranially infected with CVS11 at a dose of 10^1^ FFU/mouse, and the clinical scores and survival rates were recorded daily. The Snapin-knockdown mice exhibited milder clinical symptoms after virus infection, compared to those of control mice (Fig. 8C). Eventually, 40% of the Snapin-knockdown mice survived, compared to only 20% of the mice in the control group (Fig. 8D). The TCID_50_ and qPCR results showed that compared to the control group, the virus titers were lower in the brains of Snapin-knockdown mice (Fig. 8E and F). Subsequently, we performed IHC staining on brain tissues at 9 days post-infection. The results, as shown in Fig. 8G, revealed that the knockdown of Snapin resulted in a significant inhibition of viral replication in the mouse brain. All these results indicate that Snapin is essential for RABV infection *in vivo*.

## DISCUSSION

Although RABV infection ultimately leads to neurodegenerative changes, including neuronal apoptosis, axon degeneration, and synaptic dysfunction after entering the clinical stage, studies have demonstrated that maintaining host neuronal and axonal function and integrity is a critical prerequisite for establishing early and effective infection for neurotropic viruses. It has been reported that RABV has evolved multiple immune escape strategies, such as inhibiting the type I interferon signaling pathway via the P protein (9) and modulating the balance of Bcl-2 family proteins by M protein (13) to block the mitochondrial apoptosis pathway, thereby delaying the programmed death of infected neurons. However, many mechanisms within the molecular regulatory network governing the dynamic interactions between RABV and neurons remain to be elucidated. Here, we found a significant upregulation of Snapin following RABV infection in mouse neuron N2a cells. The overexpression of Snapin markedly enhanced RABV replication, while the knockdown of Snapin significantly reduced RABV replication in N2a cells, indicating that Snapin was involved in the replication of RABV (Fig. 1).

Snapin protein has been shown to be involved in the replication of various other viruses, but the mechanisms of action differ among different viral infections. Snapin inhibits human cytomegalovirus (HCMV) by binding UL70 to impair nuclear import and DNA synthesis, while it facilitates deformed wing virus (DWV) replication in honeybees via VP2 interaction (26, 40). These studies suggest that Snapin can either inhibit or promote viral replication depending on the specific virus and its interaction with viral proteins. Here, Co-IP and confocal results showed that Snapin could interact with RABV P proteins from both street strain (DRV) and fixed strains (CVS11 and SRV9) (Fig. 2; Fig. S2). Structural mapping revealed that the C-terminal region (172–297 amino acids) of the RABV P protein and the 37–78 amino acid region of Snapin are key domains for their interaction (Fig. 3 and 4). Notably, the C-terminal region of the RABV P protein is key for its interaction with STATs and affects the IFN pathway (41, 42); hence, at first, we assumed that Snapin might boost virus replication via regulating the interferon response. However, qPCR results showed that the changes in IFN downstream signaling factors by the infected virus after Snapin overexpression were not significant (Fig. S5).

Subsequently, through transcriptomic analysis, we found that the genes regulated by Snapin were mainly concentrated in the NF-κB signaling pathway (Fig. 5). The NF-κB signaling pathway plays a crucial role in regulating the host immune response to viral infection, particularly in inflammatory responses. Studies have shown that after viral infection, activation of the NF-κB signaling pathway leads to the production of pro-inflammatory cytokines such as IL-1, TNF-α, and IL-6 to inhibit viral replication (43, 44). These cytokines help recruit immune cells to the site of infection and initiate inflammatory responses to control viral replication. At the same time, activation of the NF-κB signaling pathway triggers the expression of antiviral genes, including IFNs and ISGs, which help inhibit viral replication and spread within host cells (45, 46). Nevertheless, some viruses are capable of modulating NF-κB in a biphasic fashion to optimize viral replication within infected cells. For example, viruses including HIV-1, EBV, HSV, and KSHV depend on the activation of the NF-κB signaling pathway to promote viral replication (47–50). In our study, we revealed that following RABV infection, the RABV P protein interacts with the host protein Snapin, which activates the NF-κB signaling pathway and thus promotes viral replication (Fig. 5E). Furthermore, we found that during RABV infection, the activation of the NF-κB signaling pathway mediated by the interaction between Snapin and P did not activate the expression of downstream cytokines (Fig. S4A). Instead, it upregulates genes related to neuronal development and synaptic stability, including *Septin2*, *B4gat1*, and *Disc1*, thus maintaining neuronal stability and providing favorable conditions for RABV replication (Fig. 5H and I). This mechanism reveals that the NF-κB signaling pathway plays a role in promoting RABV replication in a way different from that of other viruses.

However, current studies have not yet explored the direct involvement of Snapin or RABV P protein in the NF-κB signaling pathway. In this study, we utilized IP-MS and identified GSK3β as a critical linker molecule within the Snapin-P protein complex. First, we elucidated the interaction dynamics among Snapin, RABV P, and GSK3β. By employing knockout cell lines and Co-IP, we demonstrated that Snapin interacts with RABV P independently of GSK3β, whereas the interaction between GSK3β and RABV P requires Snapin (Fig. 6B and C). GSK3β, a multifunctional serine/threonine kinase highly expressed in neuronal cells, regulates diverse physiological processes, including metabolism, cell cycle progression, gene expression, development, oncogenesis, and neuroprotection (51, 52). Notably, GSK3β modulates the NF-κB signaling pathway through regulation of its kinase activity. Specifically, phosphorylation at Ser9 correlates with inhibition of its kinase activity, while phosphorylation at Tyr216 enhances kinase activity (53). Our study showed that during RABV infection, the overexpression of Snapin enhances virus replication and increases the phosphorylation levels of NF-κB p65 and GSK3β at Tyr216, while decreasing phosphorylation levels of GSK3β at Ser9. Conversely, knockdown of Snapin during RABV infection has the opposite effect (Fig. 7). These findings demonstrate that RABV P protein co-regulates the phosphorylation of GSK3β by interacting with Snapin protein, thereby activating the NF-κB signaling pathway and promoting viral replication.

Using mouse infection models, we demonstrated that Snapin expression levels correlate positively with RABV replication efficiency and neuropathogenicity in the brain. The knockdown of Snapin suppressed viral replication in the brain and improved survival rates in infected mice (Fig. 8). These results validate the proviral role of Snapin *in vivo* and propose that targeting Snapin or its downstream effectors (e.g., GSK3β) could represent a novel therapeutic strategy against RABV.

**Fig 9:**
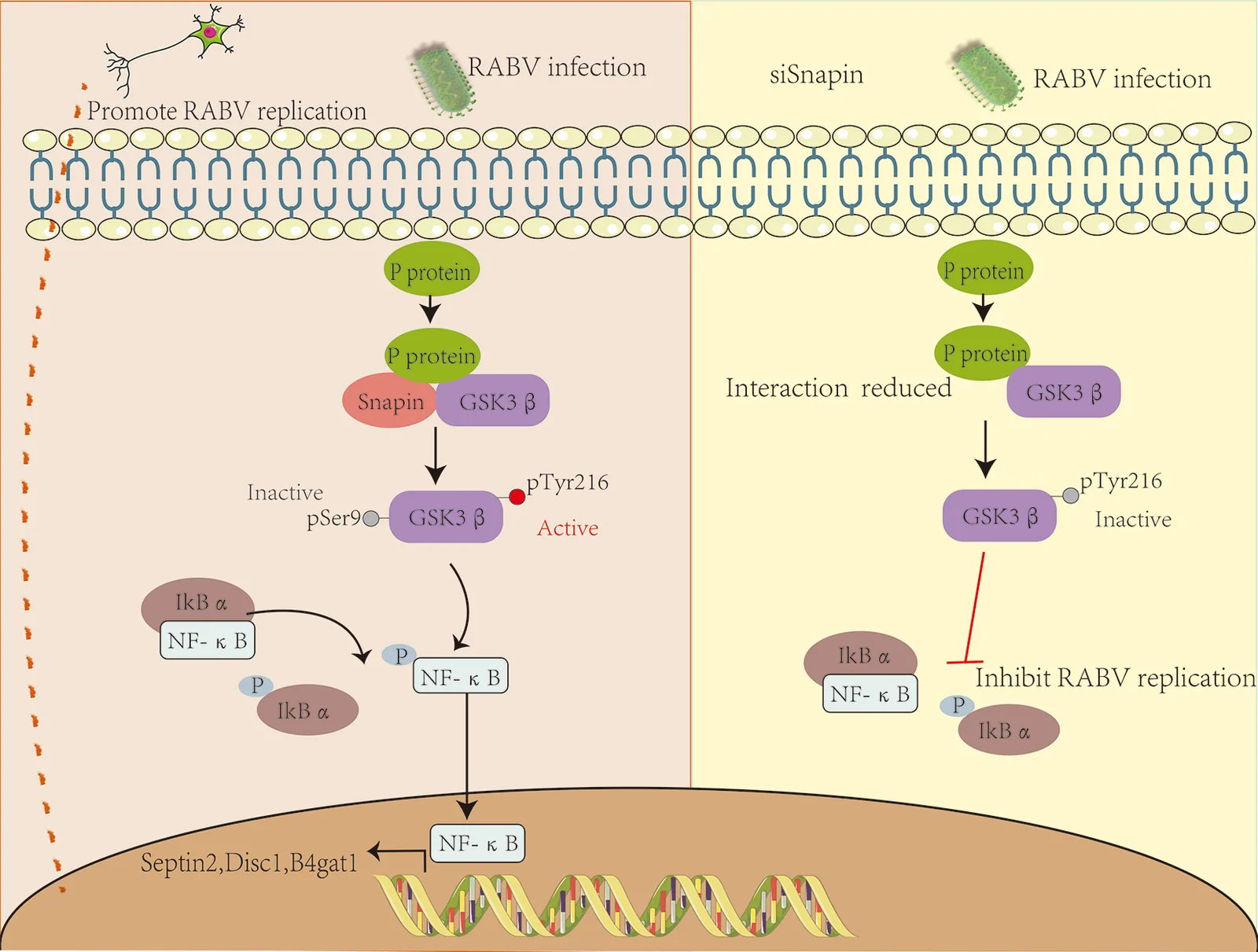
RABV P protein activates the GSK3β-NF-κB signaling pathway to promote viral replication through interacting with host protein Snapin. Upon RABV infection of host cells, the viral P protein interacts with the host protein Snapin and subsequently recruits GSK3β to form a ternary complex. This complex promotes the phosphorylation of GSK3β at Tyr216, leading to activation of the NF-κB signaling pathway. The activated pathway upregulates the expression of downstream neuron-associated genes and facilitates RABV replication. Conversely, knockdown of Snapin disrupts the GSK3β/NF-κB signaling axis and suppresses viral replication.

Taken together, in this study, we established that the host protein Snapin interacts with RABV P to activate the GSK3β/NF-κB signaling axis, altering downstream neuronal gene expression and promoting RABV replication *in vitro* and *in vivo* (Fig. 9). Additionally, as a key auxiliary SNARE protein and motor adapter in neurons, Snapin may participate in RABV replication through various intracellular transport mechanisms, beyond the GSK3β/NF-κB pathway identified here. Specifically, as a core component of the BLOC-1 complex, Snapin regulates endosomal sorting and vesicular trafficking of virus-containing compartments, thereby influencing the efficiency of viral transport from peripheral nerve endings to the neuronal cell body (19). Moreover, Snapin is involved in maintaining autophagolysosomal function; alterations in its expression may indirectly affect viral replication by modulating autophagic flux (54). Future studies should further investigate the multifaceted roles of Snapin in RABV infection, particularly its potential involvement in retrograde axonal transport of the virus.

## MATERIALS AND METHODS

### Cells, viruses, and plasmids

The N2a (mouse neuroblastoma), BSR (cloned from BHK-21), and HEK293T cell lines were generously provided by Professor Ming Zhou from Huazhong Agricultural University. The N2a, BSR, and HEK293T cells were cultured in Dulbecco’s modified Eagle’s medium (DMEM, GIBCO, USA) supplemented with 10% fetal bovine serum (GIBCO, USA), 100 units/mL penicillin, and 100 μg/mL streptomycin sulfate. Cells were grown at 37°C under an atmosphere with 5% CO_2_. Viruses (RABV CVS11 strain and SRV9 strain) were preserved in our lab (55, 56). In this study, the RABV P, M, N, and G genes used for plasmid construction were derived from the RABV CVS11 strain. The Flag-RABV P/M/N/G plasmids were constructed by cloning the gene fragments into a pcDNA3.0-based Flag vector. The Flag-RABV P truncations Flag-RABV P19–297, Flag-RABV P52–297, Flag-RABV P83–297, Flag-RABV P172–297, Flag-RABV P1–82, Flag-RABV P1–137, Flag-RABV P1–172, and Flag-RABV P1–218 were constructed by cloning the gene fragments into a pcDNA3.0-based Flag vector. HA-tagged Snapin and truncations Snapin1–37, Snapin37–78, and Snapin79–136 were constructed by inserting the gene fragments into the pcDNA3.0-based HA vector. GST-tagged Snapin and the truncations Snapin1–37, Snapin37–78, and Snapin79–136 were constructed by inserting the gene fragments into the pCAGGS-based GST vector. HA-Disc1, HA-B4gat1, and HA-Septin2 were constructed by cloning the gene fragments into a pcDNA3.0-based HA vector.

### Antibodies and reagents

Western blot, immunoprecipitation, and immunofluorescence analyses were performed using the following antibodies: Rabies virus Ab (5B12) was purchased from Novus Biologicals (Novus, USA), and GAPDH Rabbit PolyAb (10494-1-AP) and GST Tag Mouse McAb (66001-2-Ig) were purchased from Proteintech. HA Tag Monoclonal Antibody (26183), MAP2 Rabbit Ab (4542S), P-GSK-3 α/β (S21/9) Rabbit Ab (9331S), P-IKK-α/β (S176/180) (16A6) Rabbit mAb, and P-NF-κB p65 (S536) (93H1) Rabbit mAb (3033S) were purchased from CST. GSK3β (27C10) Rabbit mAb (9315S) was purchased from Invitrogen. NF-κB p65 monoclonal antibody (YM3111) was purchased from ImmunoWay. Rabbit mAb to IKKα (Y463) was purchased from Abcam. Anti-Flag antibody produced in Rabbit (F7425) was purchased from Sigma. Anti-HA antibody produced in Rabbit (H6908) was purchased from Merck. ABM Anti-Flag VHH Magarose (MA101) was purchased from ABMagic.

### Virus titration

The viral titration assay was performed using direct fluorescent antibody (DFA) in N2a cells. In a 96-well cell culture plate, the N2a cells were exposed to successive 10-fold dilutions of the viral supernatant for 48 h at 37°C. After fixation with cold acetone (80%) for half an hour at room temperature, the cells were washed thrice in phosphate-buffered saline (PBST). The cells were stained with FITC anti-rabies monoclonal globulin (1:200) and Evans blue (1:500) at 37°C for 1 h, followed by three rinses with PBST. Positive spots were identified using fluorescence microscopy (OLYMPUS, Japan), and viral titers were determined using the Karber method.

### RNA interference

All small interference RNAs (siRNAs) against *Snapin*, *B4gat1*, *Disc1*, *Septin2*, and the siRNA-negative control (NC) were designed and synthesized by Sangon Biotech (Shanghai, China). The sgRNAs used are listed in Table S1. N2a cells were seeded in six-well plates at 3 × 10^5^ cells/well, and adherent cells were transfected with the indicated siRNAs at a final concentration of 25 pmol using Lipofectamine RNAiMAX Reagent (Invitrogen, USA) for 24 h, according to the manufacturer’s recommendations. The knockdown efficiency was analyzed with quantitative real-time PCR or western blot analysis.

### qPCR

Total RNA was isolated from the cell sample using TRIzol reagent (3 × 10^6^ cell/500 μL) according to the manufacturer’s instructions (TIANGEN, China). In brief, after cracking the sample, 1/5 volume of chloroform was added, and the samples were centrifuged at 4°C and 12,000 rpm for 10 min. Then, an equal volume of isopropanol was added to the aqueous layer, and the samples were centrifuged again. The precipitate was washed twice with 75% ethanol (DEPC water preparation), and the RNA samples were resuspended in DEPC water. The cDNA was then reverse transcribed using the PrimeScript RT reagent Kit for qPCR (TAKARA, Japan). The standard curve was set with the positive plasmid pcDNA3.0-RABV N. The primers used are listed in Table S2. The copy number was calculated according to the generated standard curve. The expression of inflammatory factors in tissues was analyzed using relative quantitative real-time PCR. The primers used for qPCR are listed in Table S1. All qPCR experiments were performed on a StepOnePlus Real-Time PCR System (Thermo, USA).

### Western blot

Treated N2a cells were lysed in IP-lysis buffer (50 mM Hepes-NaOH [PH = 7.9], 100 mM NaCl, 50 mM KCl, 0.25% [vol/vol] NP-40, 1 mM DTT, and 1% [vol/vol] PMSF) and incubated for 10 min on ice. After centrifugation (12,000 rpm, 10 min, 4°C), cell lysates were collected and supplemented with SDS-PAGE loading buffer (5×). Protein samples were denatured and separated on a 10% SDS-PAGE gel before being transferred to 0.2-μm NC membranes (Cytiva, USA). The membranes were then blocked for 2 h at room temperature with 5% (wt/vol) non-fat dry milk. The primary antibodies used were against the RABV N protein (anti-mouse, 1:500), GAPDH (anti-rabbit, 1:1,000), NF-κB P65 (anti-mouse, 1:10,000), anti-NF-κB p65 (phospho S536) (anti-rabbit, 1:1,000), GSK3β (anti-rabbit, 1:1,000), GSK3β (α + β) (phospho Y216 + Y279) (anti-rabbit, 1:1,000), GSK3β (α + β) (phospho Ser21/9) (anti-rabbit, 1:1,000), HA (anti-rabbit, 1:1,000), Flag (anti-rabbit, 1:1,000), GST (anti-rabbit, 1:1,000), IKKα (anti-mouse, 1:1,000), and IKK (α + β) (phospho Y176 + Y180) (anti-rabbit, 1:1,000) and were incubated with the NC membranes overnight at 4°C. After incubation, membranes were washed five times with PBST and probed with HRP-conjugated goat anti-mouse (1:10,000) and goat anti-rabbit secondary antibodies (1:10,000). Then, the membranes were washed a further five times with PBST. Western blot band images were captured using a gel imaging system (Tanon, China), and the band grayscale values were quantified via densitometric analysis in ImageJ software and normalized to GAPDH. Following normalization to the control group, statistical comparisons between experimental groups were conducted.

### Immunofluorescence assay

BeyoGold 35-mm confocal dish samples were fixed with paraformaldehyde (Solarbio, China) at room temperature (RT) for 30 min, permeabilized with 0.5% Triton X-100 in PBS at RT for 5 min, and incubated with 0.1% BSA at 4°C overnight. Cells were then incubated with primary antibodies at 37°C for 1 h and incubated with FITC-conjugated secondary antibodies and/or TRITC-conjugated-secondary antibodies (Bioworld, USA) at 37°C for 1 h. Cellular nuclei were stained with DAPI (Beyotime, China) for 5 min. Fluorescence microscopy (OLYMPUS, Japan) was utilized to collect images. FITC fluorescence was observed after illumination at 488 nm with an emission long-band filter at 505–530 nm (green). Alexa Fluor 546 fluorescence was observed following excitation at 561 nm using an emission long-pass filter at 550–600 nm (red). DAPI was detected following excitation at 405 nm using an emission long-pass filter at 445–450 nm (blue). Mouse anti-HA antibody (Sigma, Germany), rabbit anti-Flag antibody (Sigma, Germany), and mouse anti-RABV N (Novus, USA) were used as primary antibodies.

### Co-IP assay

HEK293T cells were cotransfected with plasmids expressing Snapin protein and plasmids expressing RABV P protein using Polyjet transfection reagent. At 48 h post-transfection, the cells were lysed in IP-lysis buffer. Cell lysates were incubated with beads conjugated with anti-Flag (MCE, USA) or anti-HA (MCE, USA) on a flip shaker at 4°C overnight. Incubated beads were washed ten times with PBS and then analyzed with WB assays and SDS-PAGE. The SDS-PAGE gels were then subjected to mass spectrometry analysis by the Institute of Physics, Chinese Academy of Sciences.

### Construction of knockout cell lines

Based on the mouse Snapin and GSK3β reference nucleotide sequences published in the NCBI database, sgRNAs targeting the second exon of both proteins were designed using SnapGene software. The 20-base sequences upstream of the NGG PAM motif were selected (sequence details in Table S3). N2a cells were seeded in six-well plates at a density of 3 × 10⁵ cells/well. The pBluescriptSKII + U6-sgRNA (F + E) vector carrying the sgRNA was co-transfected with Cas9 into the cells. After 48 h, the cells were collected and subjected to GFP-positive cell sorting using an ultra-high-speed flow cytometry system. Single cells were isolated, expanded, and cultured. Genomic DNA was extracted from the expanded cell populations, amplified using validation primers, and sequenced for genotype confirmation.

### Construction of Snapin knockdown mice

Snapin gene knockdown mice were generated using the adeno-associated virus AAV-PHP.eB. The shRNA sequence targeting the murine Snapin gene was designed as GCGGTCAGAGAAAGCCAAGTAGAG. Four-week-old C57BL/6 mice were intracranially injected with 2 × 10^10^ vg/25 μL AAV-PHP.eB containing shSnapin or non-target shRNA. After 25 days, brain tissues were harvested from the mice, and the knockdown efficiency of the *Snapin* gene was validated by qPCR.

### Animal infection

Four-week-old female C57BL/6 mice were purchased from Changsheng Bio-technology Co., Ltd. The normal mice and Snapin-knockdown mice were, respectively, injected intracranially with 10^1^ FFU of RABV CVS11 virus (*n* = 16/group). The clinical score and survival rates were monitored daily (*n* = 10/group). The clinical symptoms are evaluated using a scoring system, with 0 indicating no symptoms, 1 indicating uncoordinated movement, 2 indicating a rough coat and an arched back, 3 indicating tremor and mania, 4 indicating ataxia and paralysis, and 5 indicating death. Mouse brain tissues were taken independently at 9 days post-infection (*n* = 3/group/day) and tested to detect viral load with qPCR, TCID_50_, and immunohistochemistry (IHC) analysis (57). For the IHC staining, the brain tissues were harvested and fixed in 10% formalin for a minimum of 24 h. The fixed specimens underwent deparaffinization and rehydration using xylene and graded alcohols, followed by antigen retrieval in distilled water at 100°C for 15 min. Endogenous peroxidase activity was inhibited with 3% hydrogen peroxide in methanol for 15 min, while non-specific binding was blocked with a solution of 5% skim milk. Tissue sections were incubated with primary antibodies at 37°C for 1 h, washed thoroughly, and subsequently incubated with secondary antibodies at RT for 1 h. All images were obtained using the PANNORAMIC MIDI II automatic digital slide scanner (3DHISTECH, Budapest, Hungary). Rabbit anti-Map2 antibody (1:200, CST), mouse anti-RABV P (1:200), rabbit anti-NeuN (1:200, CST), and mouse anti-β-tubulin (1:200, CST) were used as primary antibodies.

### Statistical analysis

Quantitative data are presented as mean ± standard error of the mean (SEM) with a sample size of *n* = 3 per experimental group. For pairwise comparisons between two groups, statistical significance was assessed using paired two-tailed Student's *t*-tests. When comparing three or more groups, we performed one-way analysis of variance (ANOVA), followed by Tukey’s honestly significant difference (HSD) post-hoc tests for multiple comparisons. To control family-wise error rates in multiple testing scenarios, *P*-values were adjusted using the Bonferroni correction method. Statistical significance thresholds were defined as follows: *P < 0.05, **P < 0.01, ***P < 0.001, and ****P < 0.0001. All statistical analyses were conducted using GraphPad Prism software (version 8.0; GraphPad Software Inc., San Diego, CA, USA), with verification of test assumptions including normality and homogeneity of variances as part of the analytical workflow.

## Data Availability

The sequencing data generated in this study can be accessed in the Figshare database at https://doi.org/10.6084/m9.figshare.33276981. The data underlying this article are available in the article and in its supplemental material or will be shared upon reasonable request to the corresponding author.

## SUPPLEMENTAL MATERIAL

Supplemental material jvi.01006-26-s0001.docx Fig. S1 to S7; Tables S1 to S3.

ASM does not own the copyrights to Supplemental Material that may be linked to, or accessed through, an article. The authors have granted ASM a non-exclusive, world-wide license to publish the Supplemental Material files. Please contact the corresponding author directly for reuse.
