# Supplementary material for: RABV P protein activates the GSK3β-NF-κB signaling pathway to promote viral replication by interacting with the host protein Snapin
Source: J Virol. 2026 Sep 15;100(10):e01006-26. doi: 10.1128/jvi.01006-26 (PMC13730043; doi:10.1128/jvi.01006-26)
Supplement: Supplementary file 1 [file jvi.01006-26-s0001.docx]

**Supplemental information**

**RABV P protein activates the GSK3β-NF-κB signaling pathway to promote viral replication through interacting with the host protein Snapin**

Yujie Bai, Yongsai Liu, Xiaoyu Wu, Hongli Jin, Zhiyuan Gong, Yumeng Song, Xingqi Liu, Zihang Gao, Zhenhong Guan, Pei Huang, Yuanyuan Li, Haili Zhang*, Hualei Wang*

State Key Laboratory for Diagnosis and Treatment of Severe Zoonotic Infectious Diseases, Key Laboratory for Zoonosis Research of the Ministry of Education, Institute of Zoonosis, and College of Veterinary Medicine, Jilin University, Changchun 130062, China.

**Figure. S1** **Snapin contributes to the replication of RABV SRV9 strain.** **(A)** N2a cells were transfected with si-NC or si-Snapin for 24 h, and the knockdown efficiency of si-Snapin targeting the snapin gene in N2a cells was measured using qPCR. **(B and C)** N2a cells were transfected with an empty vector, pcDNA3.1-Snapin, si-NC or si-Snapin for 24 h, followed by infection with RABV SRV9 (MOI = 0.1) for 36 h. The transcription levels of RABV N were measured using qRT-PCR **(B)**, and the protein expression levels of RABV N were measured using WB **(C)**.

**Figure. S2** **The host protein Snapin interacts with the P proteins** **of different RABV strains.** Lysates of HEK293T cells transfected with the indicated plasmids (HA-tagged Snapin and Flag-tagged RABV P protein of different RABV strains) were subjected to anti-Flag immunoprecipitation and analyzed using WB.

**Figure. S3** **Snapin does not intrinsically affect production of type Ⅰ interferons and the NF-κB signaling pathway. (A)** N2a cells were transfected with an empty vector, pcDNA3.1-Snapin, si-NC or si-Snapin for 24 h, followed by infection with RABV CVS11 (MOI = 0.1) for an additional 36 h. Transcription levels of downstream interferon-related factors were measured using qPCR. **(B)** N2a cells were transfected with an empty vector, pcDNA3.1-Snapin, si-NC or si-Snapin for 36 h. Levels of the phospho-NF-κB-p65 protein were analyzed with WB.

**Figure. S4 The host factor Snapin mediates RABV replication through regulating the expression of neuron-related factors.** **(A)** N2a cells were transfected with pcDNA3.1 vector or pcDNA3.1-snapin, and either an siRNA targeting Snapin or a control siRNA for 24 h followed by infection with RABV CVS11 (MOI = 0.1) for an additional 36 h. The transcription levels of RABV N, CCL2, CCL5, CXCL10, TNF-α, and IL-6 were measured using qPCR in harvested N2a cells. **(B)** N2a cells were transfected with pcDNA3.1 vector or pcDNA3.1-snapin, as well as an siRNA targeting Snapin or a control siRNA for 24 h, followed by infection with RABV CVS11 (MOI = 0.1) for an additional 36 h. The above samples were collected and used for RNA-sequencing (RNA-seq) analysis, and the differentially expressed genes (DEGs) were mapped. **(C)** List of high confidence genes in the DEGs as mentioned in (B). **(D)** N2a cells were transfected with pcDNA3.1 vector or pcDNA3.1-snapin for 24 h followed by infection with RABV CVS11 (MOI = 0.1) for an additional 30 h. Cell samples were collected following 6 h of treatment with the NF-κB phosphorylation inhibitor JSH-23. The transcription levels of Disc1, B4gat1, and Septin2 were measured using qPCR in harvested N2a cells.

**Figure. S5** **Host proteins that interacted with RABV P and Snapin complexes were screened by mass spectrometry combined with Co-IP assay.** N2a cells were transfected with Snapin alone or together with RABV P protein, and cell samples were collected after 36 hours. After lysis, the samples were enriched and eluted using flag magnetic beads, separated using SDS-PAGE and analyzed with mass spectrometry. **(A)** The graph shows the high confidence genes associated with NF-κB signaling pathway. **(B)** Lysates of N2a cells transfected with the indicated plasmids (HA-tagged Snapin or HA vector, Flag-tagged RABV P and GST-tagged S100B) were subjected to anti-flag immunoprecipitation and analyzed with WB. **(C)** The GSK3β gene was knocked out in N2a cells using CRISPR-Cas9 technology and the expression of GSK3β in these cells was analyzed with WB. **(D)** The Snapin gene was knocked out in N2a cells using CRISPR-Cas9 technology, and detect Snapin expression was analyzed with WB. **(E)** N2a cells were transfected with si-NC or si-Snapin for 12 h. Flag-tagged RABV P or flag vector along with GST-tagged GSK3β were then co-transfected. Subsequently, anti-GST immunoprecipitation was carried out and the results analyzed using WB.

**Figure. S6 The Snapin-P interaction regulates the phosphorylation of GSK3β and p65.** N2a cells were co-transfected with RABV P and pcDNA3.1, pcDNA3.1-Snapin, si-NC or si-Snapin for 36h. The protein expression levels of phosphorylated NF-κB p65 and GSK3β were then measured using WB.

**Figure. S7** **Cytotoxicity of TSW119 in N2a Cells.** N2a cells were seeded into 96-well plates and treated with the GSK3β inhibitor TSW119 at concentrations ranging from 0 to 800 μM. Cytotoxicity was evaluated using the CCK-8 assay.

**Supplementary Table 1. Sequences of siRNAs used in this study.**

| **Name** | **Sequence (5′–3′)** |
| --- | --- |
| siSnapin-sense | *CUCCGGGAACAAAUUGACAACTT* |
| siSnapin-antisense | *GUUGUCAAUUUGUUCCCGGAGTT* |
| siB4gat1-sense | *CCCAAUAACCUGUUAAGGAAUTT* |
| siB4gat1-antisense | *AUUCCUUAACAGGUUAUUGGGTT* |
| siDisc1-sense | *CCCAACACUCACAGUCCAATT* |
| siDisc1-antisense | *UUGGACUGUGAGUGUUGGGTT* |
| siSeptin -sense | *GCUUCGACUGUUGAGAUUGAATT* |
| siSeptin-antisense | *UUCAAUCUCAACAGUCGAAGCTT* |

**Supplementary Table 2. Primers used for qPCR.**

| **Gene** | **Primer** | **Sequence (5′–3′)** |
| --- | --- | --- |
| RABV N | Forward | *GGGGCTATTTGGGAGAGGA* |
|  | Reverse | *ATGAGTTTGGACGGGCTTG* |

**Supplementary Table 3. Sequences of sgRNAs used in this study.**

| **Name** | **Sequence (5′–3′)** |
| --- | --- |
| Snapin sgRNA-F sense sense | *CACCGAGAAAGCCAAGTAGAGCTCC* |
| Snapin sgRNA-R | *AAACGGAGCTCTACTTGGCTTTCTC* |
| GSK3β sgRNA-F | *CACCCAAGCCAAACTTTGTGATTC* |
| GSK3β sgRNA-R | *AAACGAATCACAAAGTTTGGCTTGC* |
